# Preconception Non-criteria Antiphospholipid Antibodies and Risk of Subsequent Early Pregnancy Loss: a Retrospective Study

**DOI:** 10.1007/s43032-023-01388-5

**Published:** 2023-11-06

**Authors:** Fangxiang Mu, Mei Wang, Xianghui Zeng, Ling Liu, Fang Wang

**Affiliations:** Department of Reproductive Medicine, Lanzhou University Second Hospital, No. 82 Cuiyingmen, Chengguan District, Lanzhou, 730030 China

**Keywords:** Preconception, Non-criteria antiphospholipid antibodies, Early pregnancy loss, Anti-annexin A5

## Abstract

The aim of this study was to investigate the subsequent early pregnancy outcomes in women positive for non-criteria antiphospholipid antibodies (NC-aPLs) before pregnancy. A total of 273 patients who had experienced sporadic or recurrent pregnancy loss and had been screened for 13 NC-aPLs at preconception were recruited in this study from September 2019 to February 2022. Serum levels of NC-aPLs were measured by ELISA using specific kits. The primary outcome was early pregnancy loss, and the secondary outcomes were biochemical pregnancy, clinically confirmed pregnancy loss, and ongoing pregnancy. Among these subjects, 56 patients had one previous pregnancy loss, and 217 had recurrent pregnancy loss (RPL). The NC-aPLs (+) and NC-aPLs (−) groups had similar rates of early pregnancy loss (EPL) after adjustment, regardless of the number of positive NC-aPLs (aOR = 1.054, 95% CI 0.602–1.846). Other outcomes were comparable in both groups, including the rates of biochemical pregnancy (aOR = 1.344, 95% CI 0.427–4.236), clinically confirmed pregnancy loss (aOR = 0.744, 95% CI 0.236–2.344), and ongoing pregnancy (aOR = 0.949, 95% CI 0.542–1.660). Based on sensitivity analysis, the NC-aPLs (+) were not associated with adverse early pregnancy outcomes in women with RPL. Furthermore, the difference in gestational weeks of pregnancy loss between the two groups was also insignificant. This study found no evidence linking preconception NC-aPL positivity to early pregnancy outcomes but offers a reference for future research to clarify NC-aPLs’ potential clinical impact.

**Supplementary Information:**

The online version contains supplementary material available at 10.1007/s43032-023-01388-5.

## Introduction

Antiphospholipid antibodies (aPLs) are a group of heterogeneous antibodies that target negatively charged phospholipids or negatively charged phospholipid-protein complexes, which interfere with various phospholipid-dependent coagulation and anticoagulation factors. The aPLs are associated with thrombosis formation and recurrent pregnancy failure [1, 2]. The prevalence of aPLs ranges from 1 to 7% in the general obstetric population but can reach 20% among women with a history of recurrent pregnancy loss (RPL) [3]. There is a diverse range of aPLs, including antibodies that recognize phospholipoprotein complexes (such as anti-cardiolipin (aCL)), those that directly recognize proteins (such as anti-annexin A5 (aAnxA5)), antibodies that affect phospholipid-dependent coagulation (anti-prothrombin antibody), and antibodies that directly bind to phospholipids (such as anti-phosphatidylethanolamine (aPE) antibodies) [4–7].

Therefore, the mechanisms of pathological pregnancy associated with aPLs are also complex. For example, endothelial anticoagulation dysfunction and over-activation of the complement system increase vascular permeability and promote platelet aggregation; aPL induces inflammation through TLR4/MyD88 pathway in trophoblasts, endothelial cells, and immune cells [8, 9]. These pathways lead to placental microthrombosis and inadequate remodeling of the spiral arteries, resulting in RPL, stillbirths, and other pathological pregnancies [10, 11]. Patients who tested positive for classic aPLs with vascular thrombosis or pregnancy morbidity were defined as having antiphospholipid syndrome (APS) (classic aPLs include the lupus anticoagulant (LA), aCL, and anti-β2 glycoprotein I antibodies (aβ2GPI)) [12–14]. The aPLs that do not meet the diagnosis of APS are defined as non-criteria antiphospholipid antibodies (NC-aPLs). NC-aPLs are a diverse class that includes aCL IgA, aβ2GPI IgA, aβ2GPI-domain 1 (aβ2GPI-D1), anti-phosphatidylethanolamine (aPE), anti-vimentin/cardiolipin (aVim/CL), anti-annexin A2 (aAnxA2), anti-annexin A5 (aAnxA5), and anti-phosphatidylserine/prothrombin (aPS/PT) antibodies [15–22]. There is evidence that the presence of anti-protein S (aPS) can lead to acquired PS deficiency, which was linked to RPL and fetal loss after 22 weeks [23]. In addition, anti-protein C (aPC) levels are associated with thrombosis [24, 25]. However, there was little or no evidence that aPC is associated with pregnancy loss [25].

There is substantial evidence supporting the relationship between NC-aPLs and adverse pregnancy outcomes, particularly early pregnancy loss (EPL) that occurs before 10 weeks of gestation. In a Chinese cohort study, 192 patients with APS, 90 patients with seronegative APS (SN-APS), 193 patients with autoimmune diseases, and 120 healthy subjects were compared, and at least one NC-aPL was detected in 60.9% of SN-APS and 93.5% of APS patients [26]. Furthermore, a retrospective analysis validated the association of aPS/PT with EPL, late-term abortions, and preterm delivery [27]. However, these studies mainly focused on APS patients or patients who met the clinical diagnostic criteria for APS, and did not consider women with a history of miscarriage. Therefore, it is unclear whether pre-pregnancy NC-aPLs will have adverse effects on the pregnancy outcomes in this group of patients.

In this study, we analyzed whether preconception NC-aPLs were associated with subsequent pregnancy loss in patients with a history of pregnancy loss (one or more). The secondary aim was to compare the differences in the specific time of pregnancy loss between the NC-aPL (+) and NC-aPL (−) groups.

## Materials and Methods

### Study Population

**Fig. 1:**
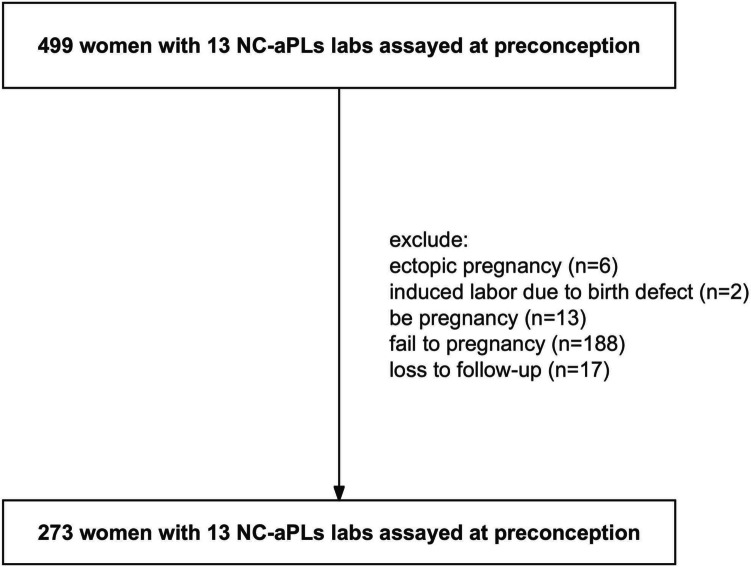
Flow chart showing recruitment of subjects

Female patients who had experienced sporadic or recurrent pregnancy loss were recruited at the Reproductive Medicine Center of the Lanzhou University Second Hospital from September 2019 to February 2022. A total of 499 patients had been screened for the 13 NC-aPLs at preconception, and 273 were included (Fig. 1) in the study based on the following criteria: (1) age over 18 years, (2) history of at least one pregnancy loss, and (3) tested for aCL IgA, aβ2GPI IgA, aβ2GPID1, aPE, aPT IgG/M, aPS/PT IgG/M, aAnxA2&5, aPC, aPS, and aVim/CL antibodies. The exclusion criteria were as follows: (1) lack of follow-up data, (2) no pregnancy outcomes prior to 10 weeks of gestation, (3) abnormal karyotypes in parents, (4) criteria aPLs positivity (aCL, or aβ2GPI, or LA), (5) uterine abnormalities, (6) late pregnancy loss (≥10 weeks), and (7) termination of pregnancy due to molar pregnancy, ectopic pregnancy, or severe birth defects.

The maternal age, body mass index (BMI), education, ethnicity, history of menstruation, previous pregnancy loss, previous live births, previous labor abortions, age at first pregnancy, and type of pregnancy loss were recorded for each patient. The study protocol was approved by the Ethics Committee of Lanzhou University Second Hospital (Approval number: No. 2019A-321). In addition, written informed consent was obtained from each patient.

### NC-aPL Evaluation

Serum levels of aβ2GPI IgG/M, aCL IgA, aβ2GPI IgA, aβ2GPID1, aPE, aPT IgG/M, aPS/PT IgG/M, aAnxA2, aAnxA5, aPC, aPS, and aVim/CL were measured by ELISA using specific kits (Aesku Diagnostics, Wendelsheim, Germany) according to the manufacturer’s instructions (Supplementary Table 1). The sera were incubated in the 96-well microplates provided in the ELISA kits for 30 min at room temperature. After washing the plates once, the substrate was added to induce enzymatic colorimetric reactions. The target antibody concentration was determined by comparing the OD (at 450 nm) of the sample to that of a standard curve. For all tests, the coefficients of variation (CV) were between 10 and 15%.

### Outcome Assessment

The primary outcome was EPL, defined as pregnancy loss before 10 weeks of gestation and including biochemical pregnancy. Secondary outcomes were biochemical pregnancy (elevated hCG level without a gestational sac on ultrasound), clinically confirmed pregnancy loss (drop in hCG levels and stalled embryonic development), and ongoing pregnancy (ultrasound showing a heartbeat in an intrauterine gestational sac after 10 weeks). RPL was defined as two and more pregnancy losses before 24 weeks of gestation, including biochemical pregnancy [28].

### Statistical Analysis

The demographic variables of the NC-aPLs (+) and NC-aPLs (−) groups were compared using Pearson’s chi-square test, Fisher’s exact test, and two sample *t*-tests. The pregnancy outcomes of the two groups were compared by Pearson’s chi-square test or Fisher’s exact test. The odds ratios (OR) were calculated using logistic models and adjusted for prior losses, maternal age, and maternal BMI.

The missing data was processed by the multivariate imputation by chained equations (MICE) algorithm in R Software (version 4.1.3). Logistic regression models were used for the binary variables (menstrual cycle, flow volume, previous history of disease), and linear regression models were used for the continuous variables (age, BMI, previous pregnancy loss, age at first pregnancy). Kaplan-Meier curves were plotted for the first trimester to estimate the cumulative incidence and gestational age of early pregnancy losses. Pregnancy loss was defined as events, and patients with ongoing pregnancy were censored regardless of late pregnancy loss, stillbirth, or live birth.

Additional sensitivity analyses were performed to assess the risks of preconception NC-aPLs, independent of the number of pregnancy losses, after excluding women with one previous pregnancy loss. In all analyses, a *P* value < 0.05 was considered statistically significant.

## Results

### Population Characteristics

A total of 499 women with a history of pregnancy loss were tested for 13 NC-aPLs at preconception, of which 273 (54.7%) met the inclusion criteria (Fig. 1), including 56 with one sporadic pregnancy loss and 217 with RPLs. Among the women with RPL, 138 had experienced two pregnancy losses, 58 had three pregnancy losses, and 21 had four or more pregnancy losses. The demographic characteristics of both groups are summarized in Table 1 and Supplementary Table 2. No statistically significant differences were observed in any of the demographic characteristics.

**Table 1:** Demographic characteristics of participants in different patient groups

Characteristics	NC-aPLs (−) *n* = 141	NC-aPLs (+) *n* = 132	*P*-value
Age (years)	30.22 ± 3.20	30.36 ± 4.31	0.754
BMI (kg/m^2^)	21.91 ± 2.95	22.07 ± 3.36	0.673
Previous labor abortion			0.289
0	125 (88.65)	122 (92.42)	
≥1	16 (11.35)	10 (7.58)	
Previous live births			0.930
0	117 (82.98)	109 (82.58)	
≥1	24 (17.02)	23 (17.42)	
Previous pregnancy loss			0.538
1	26 (18.44)	30 (22.73)	
2	77 (54.61)	61 (46.21)	
3	27 (19.15)	31 (23.48)	
≥4	11 (7.80)	10 (7.58)	
Type of pregnancy loss			0.929
Primary	93 (80.87)	82 (80.39)	
Secondary	22 (19.13)	20 (19.61)	
Previous history of disease			0.221
None	136 (96.45)	123 (93.18)	
Diseases	5 (3.55)	9 (6.82)	
Previous history of surgery			0.477
None	121 (85.82)	106 (80.30)	
Gynecologic and obstetric	17 (12.06)	22 (16.67)	
Other surgery	3 (2.13)	4 (3.03)	

Data are shown as mean ± standard deviation, or frequency with percentages

*BMI* body mass index

### Association of Preconception NC-aPLs with Early Pregnancy Outcomes

**Fig. 2:**
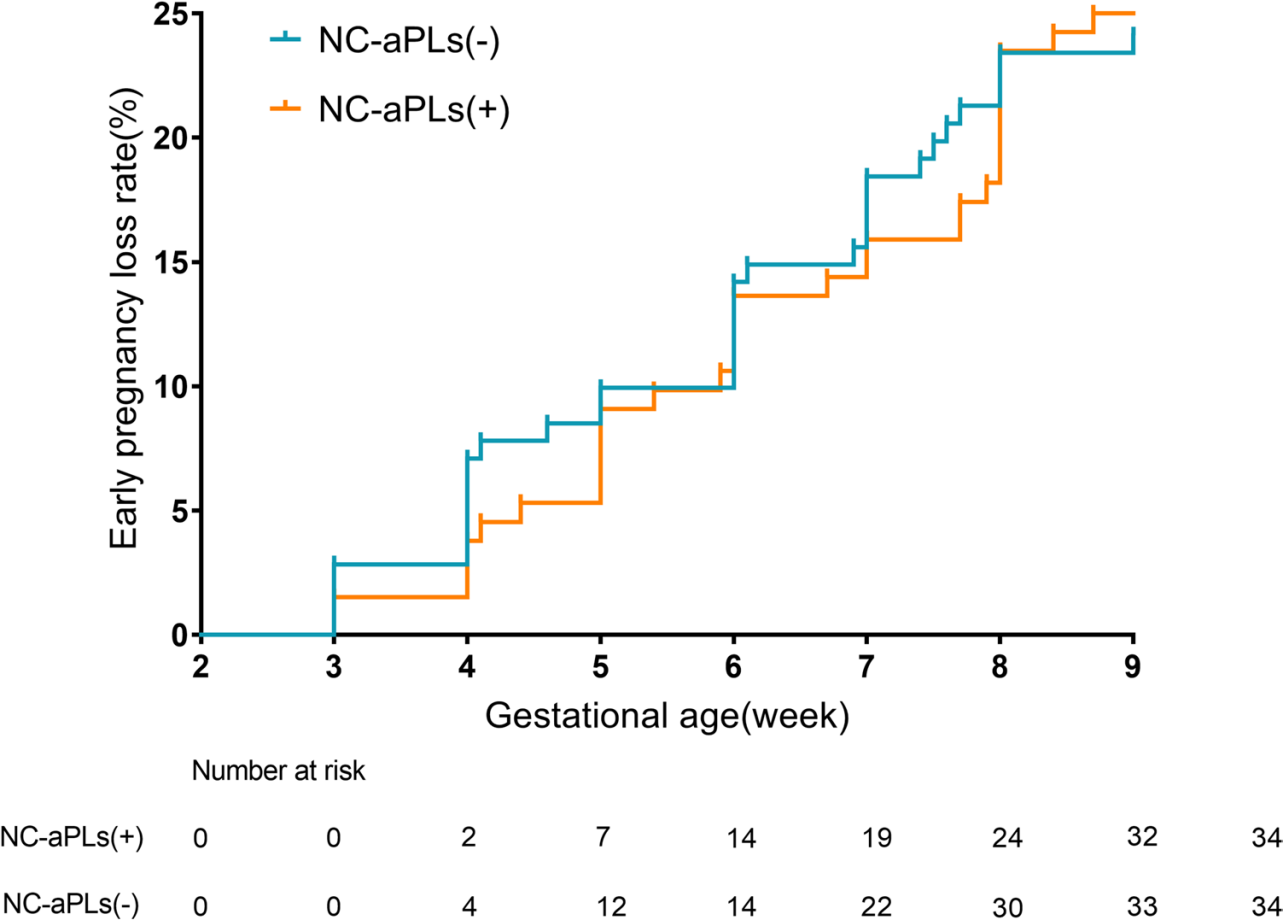
Kaplan-Meier curves of the NC-aPLs (+) and NC-aPLs (−)

Overall, 205 subjects (75.1%) had an ongoing pregnancy. EPL and biochemical pregnancy, respectively, accounted for 24.9% (68/273) and 6.2% (17/273) of the pregnancy losses. In addition, 34/132 (25.8%) NC-aPLs (+) patients experienced EPL compared to 34/141 (24.1%) NC-aPLs (−) patients (*P* = 0.754; Supplementary Table 3). The NC-aPLs were not associated with early pregnancy outcomes even after adjusting for menstrual cycle, flow volume, previous history of disease, age, BMI, previous pregnancy loss, and age at first pregnancy. Any positive status was defined by the presence of any antibody, and no significant difference was observed in the rate of EPL between the NC-aPLs (+) and NC-aPLs (−) groups (aOR = 1.054, 95% CI 0.602–1.846). There was no significant difference in the rate of biochemical pregnancy between the two groups after adjusting for the confounding factors, regardless of the presence of one, two, or multiple NC-aPLs (aOR = 1.344, 95% CI 0.427–4.236). Other secondary outcomes, including the frequency of clinically recognized pregnancy loss (aOR = 0.744, 95% CI 0.236–2.344) and ongoing pregnancy rate (aOR = 0.949, 95% CI 0.542–1.660) were also similar between the NC-aPLs (+) and NC-aPLs (−) groups (Table 2). As shown in Supplementary Table 4, the prevalence of aPT was highest (14.3%), followed by that of aAnxA5 (11.0%), aβ2GPID1 (9.9%), aPC (8.8%), and aPE (7.3%) in all patients. The cumulative incidence of early pregnancy loss was similar in NC-aPLs (+) and NC-aPLs (−) patients (Fig. 2).

**Table 2:** Correlations of preconception NC-aPLs and pregnancy outcomes in women experience pregnancy loss

	Any positive		Any two positive		Multiple positive	
	Unadjusted	Adjusted	Unadjusted	Adjusted	Unadjusted	Adjusted
Early pregnancy loss	1.092 (0.631–1.890)	1.054 (0.602–1.846)	0.542 (0.250–1.178)	0.540 (0.245–1.190)	0.448 (0.098–2.036)	0.463 (0.100–2.154)
Biochemical pregnancy	0.855 (0.285–2.566)	1.344 (0.427–4.236)	0.838 (0.157–4.484)	1.132 (0.207–6.209)	-	-
Clinically recognized pregnancy loss	1.170 (0.390–3.512)	0.744 (0.236–2.344)	1.193 (0.223–6.384)	0.883 (0.161–4.841)	-	-
Ongoing pregnancy	0.916 (0.529–1.585)	0.949 (0.542–1.660)	1.844 (0.844–4.004)	1.854 (0.840–4.090)	2.234 (0.491–10.163)	2.159 (0.464–10.039)

Unadjusted did not adjust any variables. Adjusted was adjusted for the maternal adjustment model including menstrual cycle, flow volume, previous history of disease, age, BMI, and age at first pregnancy. Any positive status was defined by the presence of any antibody positive

*NC-aPLs* non-criteria antiphospholipid antibodies

### Sensitivity Analyses of Preconception NC-aPLs After Exclusion of Women with One Sporadic Pregnancy Loss

Additional sensitivity analyses were performed to determine the risks of preconception NC-aPLs independent of one sporadic pregnancy loss. Considering the risk of EPL in multivariate analyses, no difference was observed with regard to single-positive NC-aPL (aOR = 1.312, 95%CI 0.672–2.561), any two-positive NC-aPLs, or multiple-positive NC-aPLs. Preconception NC-aPLs were not associated with clinically confirmed pregnancy loss (aOR = 1.242, 95%CI 0.334–4.616), or ongoing pregnancies (aOR = 0.753, 95%CI 0.392–1.447) (Table 3).

**Table 3:** Correlations of preconception NC-aPLs and pregnancy outcomes in patients with recurrent pregnancy losses

	Any positive		Any two positive		Multiple positive	
	Unadjusted	Adjusted	Unadjusted	Adjusted	Unadjusted	Adjusted
Early pregnancy loss	1.360 (0.737–2.511)	1.312 (0.672–2.561)	0.510 (0.213–1.225)	0.479 (0.182–1.262)	0.254 (0.032–2.014)	0.322 (0.038–2.709)
Biochemical pregnancy	0.716 (0.218–2.354)	0.805 (0.217–2.993)	1.077 (0.185–6.254)	1.286 (0.186–8.914)	-	-
Clinically recognized pregnancy loss	1.397 (0.425–4.593)	1.242 (0.334–4.616)	0.929 (0.160–5.392)	0.777 (0.112–5.388)	-	-
Ongoing pregnancy	0.735 (0.398–1.358)	0.753 (0.392–1.447)	1.959 (0.817–4.701)	2.085 (0.820–5.304)	3.934 (0.497–31.167)	3.879 (0.459–32.820)

Unadjusted did not adjust any variables. Adjusted was adjusted for the maternal adjustment model including menstrual cycle, flow volume, previous history of disease, age, BMI, and age at first pregnancy. Any positive status was defined by the presence of any antibody positive

*NC-aPLs* non-criteria antiphospholipid antibodies

## Discussion

In the present study, we explored early pregnancy outcomes in women with positive NC-aPLs before pregnancy and did not find a correlation between the two variates. In addition, our findings indicated that the presence of NC-aPLs was not associated with the gestational week of EPL (including biochemical pregnancy and clinically confirmed pregnancy loss), or with early pregnancy outcome (EPL or ongoing pregnancy).

Sensitivity analysis further showed that positive preconception NC-aPLs did not affect early pregnancy outcomes in women with only one pregnancy loss, possibly since the latter may also be associated with chromosomal abnormalities. The European Society of Human Reproduction and Embryology (ESHRE, 2022) identified genetic defects as an etiological factor of incidental pregnancy loss and RPL [29]. In a systematic review, the prevalence of chromosomal abnormalities in incidental pregnancy loss was 45% (95% CI 38–52; 13 studies; 7012 samples) [29, 30]. In a recent study evaluating 200 pregnancy losses, 42% of confirmed inheritance was autosomal recessive (30.8%), X-linked recessive (3.8%), or autosomal dominant (excluding neonates, 7.7%), with a risk of recurrence in future pregnancies [31]. This confounding factor was not excluded in our study since embryonic chromosome testing was not routinely performed in the patient population. Studies that exclude embryonic chromosomal abnormalities need to be conducted in future to validate our findings.

We also found that preconception NC-aPLs did not affect the outcome of subsequent pregnancies in women with a history of RPL. Zhu et al. studied the association of some NC-aPLs with women with unexplained episodic pregnancy loss or RPL. The patients were positive for aPE IgM (40.0%), aPE IgG (12.8%), and aPT IgM (10.4%), and the combined aPE IgG and anti-endometrium antibody (aEM) IgG biomarker clearly distinguished the patients with pregnancy loss [32]. Although the population was similar to that followed in our study, the researchers did not focus on the effect of these NC-aPLs on subsequent pregnancies and their results are therefore not comparable. Another inconsistency with the previous study is that aPT was the predominant antibody in our study, followed by aAnxA5. This can be attributed to differences in the detection method, detection threshold, and laboratory standards. Truglia et al. evaluated 20 pregnancies in 17 patients who met the clinical criteria for SN-APS, and found that 12 patients (60%) had a good outcome with conventional treatment [33]. However, we focused on the correlation between preconception NC-aPLs and subsequent pregnancies rather than the effect of drugs on pregnancy outcomes. Finally, the differences in laboratory testing criteria, NC-aPLs thresholds, and study population may also explain the inconsistencies between the results of our study and the previous studies.

Since biochemical pregnancies may result from chromosomal abnormalities, the outcomes may be incidental. However, there is no conclusive data at present to support this hypothesis. Maternal age-related chromosomal aneuploidy may be associated with biochemical pregnancy [34–36]. Vaiarelli et al. investigated the relationship between biochemical pregnancy and chromosomal status after frozen embryo transfer (FET) and found that biochemical pregnancy was independent of the developmental stage of the embryo, the use of trophoblast ectoderm biopsy, and the chromosomal structure of FET [37]. Further research is needed to fully understand the underlying causes of biochemical pregnancies.

Pharmacological treatment may have contributed to the similar results observed in the NC-aPL-positive and -negative groups in our study. The conventional treatment protocol for RPL is aspirin and low-molecular heparin. Antibodies can cause endothelial anticoagulation dysfunction and over-activation of the complement system, which increases vascular permeability and promotes platelet aggregation [8]. In addition, aspirin has been reported to suppress humoral immune responses by inhibiting antibody synthesis, while low-molecular heparin is a common anticoagulant [38, 39]. Abisror et al. observed a significant improvement in the cumulative incidence of adverse obstetric events in AN-APS patients treated with aspirin or the combination of aspirin and low-molecular heparin compared to the untreated group (log-rank < 0.05), whereas both treatment groups had similar frequency of adverse obstetric events [40]. Thus, our study results may be influenced by the conventional treatment of RPL, which improves subsequent pregnancy outcomes. It remains to be ascertained whether the impact of preconception NC-aPLs on subsequent pregnancies in patients with RPL is influenced by pharmacological interventions.

This study is the first to examine the association between preconception NC-aPLs and subsequent pregnancy outcomes in women with a history of pregnancy loss. Moreover, we designed this study in a relatively comprehensive manner, with 13 NC-aPLs analyzed and the impact of having 1, 2, 3, and more positive antibodies on pregnancy outcomes was also studied. In addition, our findings provide ample evidence of the importance of testing preconception NC-aPL levels. Nevertheless, there are several limitations in our study that ought to be considered. First, this was a retrospective study and it is thus potentially susceptible to recall bias. Second, all indicators were tested only once during the first 3–6 months of pregnancy, which is potentially susceptible to transient positivity. Although baseline variables and covariates were adjusted, we cannot rule out the impact of some confounding variables, such as advanced age, thyroid disorder, insulin resistance, and psychological health factors. These factors may add complexity and uncertainty to the results. For our future research, we aim to improve our study design by increasing the sample size and implementing more rigorous inclusion and exclusion standards. This will allow us to thoroughly examine the effect of NC-aPLs on the subsequent pregnancy outcomes of women who have experienced pregnancy loss. Therefore, well-designed randomized controlled trial studies are needed to further elucidate the relationship between preconception NC-aPLs and subsequent pregnancy outcomes. Additionally, this was a single-center study, and more comprehensive analysis using data from other centers will thus be necessary to validate our findings.

In conclusion, this study investigated the association of preconception NC-aPLs with early pregnancy outcomes in women with a history of pregnancy loss. Preconception NC-aPLs had no impact on pregnancy outcomes (including biochemical pregnancy, clinically confirmed pregnancy loss, and ongoing pregnancy). In addition, there was no significant difference in the gestational week of pregnancy loss between the NC-aPLs (+) and NC-aPLs (−) groups. In this study, preconception NC-aPLs positivity did not have statistically significant effects on early pregnancy outcomes; however, it provides a valuable reference for future research aimed at elucidating the potential clinical impact of NC-aPLs.

### Supplementary Information

ESM 1 (DOCX 17 kb)

ESM 2 (DOCX 17 kb)

ESM 3 (DOCX 15 kb)

ESM 4 (DOCX 15 kb)
