# Supplementary material for: Preconception Non-criteria Antiphospholipid Antibodies and Risk of Subsequent Early Pregnancy Loss: a Retrospective Study
Source: Reprod Sci. 2023 Nov 6;31(3):746–53. doi: 10.1007/s43032-023-01388-5 (PMC10912122; doi:10.1007/s43032-023-01388-5)
Supplement: Supplementary file 1 — (DOCX 17 kb) [file 43032_2023_1388_MOESM1_ESM.docx]

**Supplementary Table 1.** Antibody information

| **Antibodies full name** | **Antibodies abbreviation** | **Detection method** | **Assay kit** | **Instrument** | **Interval/ Threshold** |
| --- | --- | --- | --- | --- | --- |
| Anti-cardiolipin antibody-IgG | aCL-IgG | ELISA | Ruipin biology | Sunrise TECAN | Negative value \|\|<12.00 GPL/mL  Cut off value\|\|12.00-18.00 GPL/mL  Positive value \|\|>18.00 GPL/mL |
| Anti-cardiolipin antibody-IgM | aCL- IgM | ELISA | Ruipin biology | Sunrise TECAN | Negative value \|\|<12.00 MPL/ML  Cut off value\|\|12.00-18.00 MPL/ML  Positive value \|\|>18.00 MPL/ML |
| Anti-cardiolipin antibody-IgA | aCL-IgA | ELISA | Ruipin biology | Sunrise TECAN | Negative value \|\|<12.00 APL/ML Cut off value\|\|12.00-18.00 APL/ML  Positive value \|\|>18.00 APL/ML |
| Anti-β2 Glycoprotein I-IgG | aβ2GPⅠ- IgG | ELISA | Ruipin biology | Sunrise TECAN | Negative value \|\|<12.00 U/mL  Cut off value\|\|12.00-18.00 U/mL  Positive value \|\|>18.00 U/mL |
| Anti-β2 Glycoprotein I-IgM | aβ2GPⅠ- IgM | ELISA | Ruipin biology | Sunrise TECAN |  |
| Anti-β2 Glycoprotein I-IgA | aβ2GPⅠ-IgA | ELISA | Ruipin biology | Sunrise TECAN |  |
| Lupus anticoagulants | LA | HemosIL Silica Clotting Time | STAGO | Stago-Compact Max | LA1: 28.6-59.4s  LA2: 36.6-57.2s |
| Anti-β2Glycoprotein I domain 1 | aβ2GPI-D1 | ELISA | Ruipin biology | Sunrise TECAN | ≤65.00ng/mL |
| Anti-Annexin A2 | aAnxA2 | ELISA | Ruipin biology | Sunrise TECAN | ≤100.00 ng/mL |
| Anti-Annexin A5 | aAnxA5 | ELISA | Ruipin biology | Sunrise TECAN | ≤60.00 ng/mL |
| Anti-prothrombin-IgM | aPT-IgM | ELISA | AESKU | Sunrise TECAN | Negative value \|\|<12.00  Cut off value\|\|12.00-18.00  Positive value \|\|>18.00 |
| Anti-prothrombin-IgG | aPT-IgG | ELISA | AESKU | Sunrise TECAN | Negative value \|\|<12.00  Cut off value\|\|12.00-18.00  Positive value \|\|>18.00 |
| Anti-protein C | aPC | ELISA | Ruipin biology | Sunrise TECAN | ≤210.00 ng/mL |
| Anti-protein S | aPS | ELISA | Ruipin biology | Sunrise TECAN | ≤135.00 ng/mL |
| Anti-vimentin/cardiolipin | aVim/CL | ELISA | Ruipin biology | Sunrise TECAN | ≤90.00 ng/mL |
| Anti-phosphatidylserine/prothrombin-IgM | aPS/PT-IgM | ELISA | INOVA | Sunrise TECAN | 0.00-30.00 U/mL |
| Anti-phosphatidylserine/prothrombin-IgG | aPS/PT-IgG | ELISA | INOVA | Sunrise TECAN | 0.00-30.00 U/mL |
| Anti-phosphatidylethanolamine | aPE | ELISA | Ruipin biology | Sunrise TECAN | ≤70.00 ng/mL |
