# Supplementary material for: Preconception Non-criteria Antiphospholipid Antibodies and Risk of Subsequent Early Pregnancy Loss: a Retrospective Study
Source: Reprod Sci. 2023 Nov 6;31(3):746–53. doi: 10.1007/s43032-023-01388-5 (PMC10912122; doi:10.1007/s43032-023-01388-5)
Supplement: Supplementary file 2 — (DOCX 17 kb) [file 43032_2023_1388_MOESM2_ESM.docx]

**Supplementary Table 2.** Demographic characteristics of participants in different patient groups

| **Characteristics** | **NC-aPLs (-)**  **n = 141** | **NC-aPLs (+)**  **n = 132** | ***P*-value** |
| --- | --- | --- | --- |
| Age of menarche (years) | 13.60 ± 1.29 | 13.48 ± 1.32 | 0.457 |
| Age at first pregnancy (years) | 26.36 ± 3.26 | 26.54 ± 3.56 | 0.670 |
| Education |  |  | 0.886 |
| Primary and illiteracy | 3 (2.13) | 2 (1.52) |  |
| Middle school | 18 (12.77) | 18 (13.64) |  |
| High school | 21 (14.89) | 17 (12.88) |  |
| University | 92 (65.25) | 91 (68.94) |  |
| Postgraduate and higher | 7 (4.96) | 4 (3.03) |  |
| Ethnicity |  |  | 0.999 |
| Han Chinese | 129 (91.49) | 120 (90.91) |  |
| Hui Chinese | 6 (4.26) | 6 (4.55) |  |
| Zang Chinese | 3 (2.13) | 3 (2.27) |  |
| Other ethnic groups | 3 (2.13) | 3 (2.27) |  |
| Regularity |  |  | 0.323 |
| Regular | 118 (83.69) | 116 (87.88) |  |
| Irregular | 23 (16.31) | 16 (12.12) |  |
| Menstrual cycle |  |  | 0.453 |
| Normal | 116 (82.27) | 105 (79.55) |  |
| Infrequent or Absent | 10 (7.09) | 15 (11.36) |  |
| Other | 15 (10.64) | 12 (9.09) |  |
| Flow volume |  |  | 0.186 |
| Light | 65 (46.10) | 47 (35.61) |  |
| Normal | 71 (50.35) | 81 (61.36) |  |
| Heavy | 5 (3.55) | 4 (3.03) |  |
| Pain scale of Dysmenorrhea |  |  | 0.691 |
| None | 60 (42.55) | 49 (37.12) |  |
| Light | 64 (45.39) | 62 (46.97) |  |
| Obvious | 11 (7.80) | 15 (11.36) |  |
| Heavy | 6 (4.26) | 6 (4.55) |  |

**Note:** Data are shown as mean ± standard deviation, or frequency with percentages. BMI, body mass index.
