# Supplementary material for: Preconception Non-criteria Antiphospholipid Antibodies and Risk of Subsequent Early Pregnancy Loss: a Retrospective Study
Source: Reprod Sci. 2023 Nov 6;31(3):746–53. doi: 10.1007/s43032-023-01388-5 (PMC10912122; doi:10.1007/s43032-023-01388-5)
Supplement: Supplementary file 3 — (DOCX 15 kb) [file 43032_2023_1388_MOESM3_ESM.docx]

**Supplementary Table 3.** Outcome of Subsequent Pregnancies in Different NC-aPLs Status During Preconception

|  | | Any Positive | | | Any Two Positive | | | | Multiple Positive | | | | |
| --- | --- | --- | --- | --- | --- | --- | --- | --- | --- | --- | --- | --- | --- |
|  | + | - | *P*-value | + | | - | *P*-value | + | | - | | | *P*-value |
|  | n = 132 | n = 141 |  | n = 54 | | n = 219 |  | n = 15 | | | n = 258 |  | |
| Early Pregnancy Loss | 34 (25.8) | 34 (24.1) | 0.754 | 9 (16.7) | | 59 (26.9) | 0.118 | 2 (13.3) | | | 66 (25.6) | | 0.286 |
| Biochemical Pregnancy | 8 (22.9) | 9 (26.5) | 0.779 | 2 (22.2) | | 15 (25.4) | >0.99 | 0 (0.0) | | | 17 (25.5) | | >0.99 |
| Clinically Recognized Pregnancy loss | 26 (74.3) | 25 (73.5) | 0.779 | 7 (77.8) | | 44 (74.6) | >0.99 | 2 (66.7) | | | 49 (74.2) | | >0.99 |
| Ongoing Pregnancy | 98 (74.2) | 107 (75.9) | 0.754 | 45 (83.3) | | 160 (73.1) | 0.118 | 13 (86.7) | | | 192 (74.4) | | 0.286 |

**Note:** Data are expressed as number with percentages. NC-aPLs, non-criteria antiphospholipid antibodies.
