# Supplementary material for: Preconception Non-criteria Antiphospholipid Antibodies and Risk of Subsequent Early Pregnancy Loss: a Retrospective Study
Source: Reprod Sci. 2023 Nov 6;31(3):746–53. doi: 10.1007/s43032-023-01388-5 (PMC10912122; doi:10.1007/s43032-023-01388-5)
Supplement: Supplementary file 4 — (DOCX 15 kb) [file 43032_2023_1388_MOESM4_ESM.docx]

**Supplementary Table 4.** Positive rates of the non-criteria antiphospholipid antibodies in different patient groups

| **NC-aPLs** | **One sporadic pregnancy loss**  **n = 56** | **Recurrent pregnancy loss**  **n = 217** | ***P*-value** |
| --- | --- | --- | --- |
| aCL IgA | 0 (0) | 1 (0.5) | 1.000^b^ |
| aβ2GP1 IgA | 2 (3.6) | 1 (0.5) | 0.203^b^ |
| aβ2GP1D1 | 7 (12.5) | 20 (9.2) | 0.463^a^ |
| aAnxA2 | 4 (7.1) | 12 (5.5) | 0.889^b^ |
| aAnxA5 | 8 (14.3) | 22 (10.1) | 0.376^a^ |
| aPT IgG | 3 (5.4) | 16 (7.4) | 0.815^b^ |
| aPT IgM | 5 (8.9) | 15 (6.9) | 0.819^b^ |
| aPC | 4 (7.1) | 20 (9.2) | 0.823^b^ |
| aPS | 2 (3.6) | 16 (7.4) | 0.471^b^ |
| aVim/CL | 1 (1.8) | 13 (6.0) | 0.351^b^ |
| aPS/PT IgG | 1 (1.8) | 2 (0.9) | 1.000^b^ |
| aPS/PT IgM | 3 (5.4) | 6 (2.8) | 0.583^b^ |
| aPE | 4 (7.1) | 16 (7.4) | 1.000^b^ |

**Note:** Data are expressed as number with percentages. aCL, anti-cardiolipin; aβ2GP1, anti-β2GPI; aβ2GPI-D1, anti-β2GPI-domain1; aAnxA2, anti-annexin A2; aAnxA5, anti-annexin A5; aPT, anti-prothrombin; aPC, anti-protein C, aPS, anti-protein S; aVim/CL, anti-vimentin/cardiolipin; aPS/PT, anti-phosphatidylserine/prothrombin; aPE, anti-phosphatidylethanolamine antibodies; NC-aPLs, non-criteria antiphospholipid antibodies.

^a^ Analysis by Chi-square test;

^b^ Analysis by correct Chi-square test
